# How anticipated positive and negative emotions influence pro-environmental behavior via environmental attitudes

**DOI:** 10.3389/fpsyg.2025.1625619

**Published:** 2025-08-14

**Authors:** Xueting Niu, Menghan Li, Zhenhe Sun, He Li

**Affiliations:** ^1^School of Public Administration, Northwest University, Xi’an, China; ^2^School of Emergency Management, Northwest University, Xi’an, China

**Keywords:** anticipated emotions, pro-environmental behavior, environmental attitudes, theory of planned behavior, affective events theory

## Abstract

**Background:**

Anticipated emotions are important predictors of pro-environmental behavior within the extended theory of planned behavior. However, their mechanisms need further exploration through emotion theories. This study applies Affective Events Theory (AET), a framework originally developed to explain emotional influences on behavior in workplace settings and more recently extended to the context of pro-environmental behavior, to examine how anticipated positive and negative emotions influence pro-environmental behavior, with environmental attitudes serving as a mediator.

**Methods:**

A cross-sectional survey was conducted to measure anticipated positive and negative emotions, environmental attitudes, pro-environmental behavior, and demographic variables. A total of 500 questionnaires were randomly distributed via the Credamo online platform, yielding 442 valid responses.

**Results and conclusion:**

Both anticipated positive and negative emotions positively influence pro-environmental behavior, with environmental attitudes mediating these effects. Specifically, anticipated positive emotions not only exert a direct impact on pro-environmental behavior but also indirectly influence it through environmental attitudes. In contrast, anticipated negative emotions affect pro-environmental behavior exclusively through the mediating role of environmental attitudes. The direct effect of anticipated positive emotions may reflect the approach-oriented motivational function of positive emotions. Meanwhile, the indirect effects of both types of emotions through environmental attitudes suggest that anticipated emotions promote pro-environmental behavior by influencing individuals’ perceptions of the human–environment relationship. These findings significantly advance our understanding of the emotional mechanisms underlying pro-environmental behavior and offer valuable implications for designing emotion-based interventions aimed at fostering environmentally responsible actions.

## Introduction

The *Global Risks Report 2024* highlights that environmental risks have consistently ranked among the top global threats over the past years, encompassing extreme weather events, pollution, biodiversity loss and ecosystem collapse, and natural resource shortages. In response to these environmental challenges, the study of pro-environmental behavior has garnered substantial attention from scholars.

Pro-environmental behavior is defined as “behavior that harms the environment as little as possible, or even benefits the environment” (Steg and Vlek, 2009). Many theories have been proposed to explain and predict individuals’ pro-environmental behavior, one of the most influential of which is the theory of planned behavior (TPB). According to TPB, individuals’ behavioral intentions and actual behaviors are primarily driven by three predictors: attitudes toward the behavior, subjective norms, and perceived behavioral control (Ajzen, 1991; Ajzen, 2002; Rivis et al., 2009). Research has consistently highlighted the importance of these factors in shaping individuals’ pro-environmental behavior (Li et al., 2019; Yuriev et al., 2020). For example, De Groot and Steg (2007) applied the TPB to examine the use of park-and-ride facilities in Groningen, the Netherlands. Their study found that attitudes toward the park-and-ride facilities, subjective norms, and perceived behavioral control were all significant predictors of individuals’ intentions to use these facilities. Similarly, Gansser and Reich (2023) explored the factors influencing individuals’ behavioral intentions toward nature conservation and environmental protection within the TPB framework. Their findings indicated that attitude, subjective norms, and perceived behavioral control positively predicted pro-environmental behavioral intentions. Subsequent research has expanded the predictors of behavior within the TPB framework, including anticipated emotions (Ajzen and Sheikh, 2013; Sandberg and Conner, 2008; Wolff et al., 2011), moral norms (Chan and Bishop, 2013; Wang et al., 2018), self-identity (White and Hyde, 2012), and demographic variables (Sniehotta et al., 2013). These extensions of TPB have significantly improved the model’s explanatory power regarding various behaviors, particularly in predicting pro-environmental behaviors (Li et al., 2019).

Anticipated emotions refer to the emotions individuals expect to feel as a result of performing or not performing a behavior (Bagozzi et al., 2016; Rivis et al., 2009), which has been shown as one of the powerful drivers of pro-environmental behavior (Lu et al., 2020; Zhang et al., 2018). For instance, Zhang et al. (2018) found that anticipated positive emotions significantly influenced urban park visitors’ intentions to engage in environmentally friendly behaviors. Lu et al. (2020) demonstrated that anticipated pride and guilt promoted employees’ pro-environmental behavior in organizations. Meta-analytic studies have further supported these findings. For example, Sandberg and Conner (2008) showed that including anticipated regret in the TPB model accounted for an additional 7% of the variance in intentions and 1% in behavior, while Rivis et al. (2009) found that anticipated affect explained an additional 5% of the variance in pro-environmental intentions.

Despite growing evidence for the predictive role of anticipated emotions in promoting pro-environmental behavior, most studies within the TPB framework have not adequately addressed the underlying mechanisms of this influence (Lu et al., 2020; Zhang et al., 2018), particularly from the perspective of emotion theories. This limitation hampers a deeper understanding of the emotional processes that drive pro-environmental behaviors.

To address this gap, the present study investigates how anticipated emotions influence pro-environmental behavior by drawing on Affective Events Theory (AET). AET proposes that individuals’ emotional responses to significant events can shape their attitudes and behaviors either directly or indirectly (Weiss and Beal, 2005; Weiss and Cropanzano, 1996). While originally developed to explain behavior in organizational settings, recent studies have shown that AET can also account for the emotional pathways underlying pro-environmental behavior (Iqbal et al., 2023; Mi et al., 2021). Compared to the TPB approach, applying AET offers a more nuanced view of how emotional anticipation shapes environmentally responsible behavior.

## Literature review and hypotheses

### Affective events theory

Weiss and Cropanzano (1996) introduced the affective events theory (AET), which posits that features of the work environment trigger work events that provoke affective reactions in individuals. These affective reactions, in turn, influence their attitudes and behaviors. Affective reactions influence behavior through two pathways: directly, by affecting employees’ behaviors, referred to as affective-driven behaviors, and indirectly, by shaping attitudes (e.g., job satisfaction), which in turn influence behavior, known as judgment-driven behaviors. Since its development, the theory has been applied in various fields, including organizational behavior, leadership, and nursing (Christensen et al., 2023; Dasborough, 2006; Zhao et al., 2007).

Recently, researchers have extended its application to the study of pro-environmental behavior in organizational settings. For instance, Mi et al. (2021) applied AET to examine environmental citizenship behavior in the workplace, finding that perceptions of environmental pollution events lead to anticipated guilt, which in turn affects employees’ organizational citizenship behaviors toward the environment. Similarly, Iqbal et al. (2023) explored the impact of environmentally specific transformational leadership on employees’ green attitudes and behaviors, showing that harmonious environmental passion mediates the positive relationship between leadership and pro-environmental attitudes and behaviors. These studies highlight the significant potential of AET in understanding and promoting pro-environmental behavior.

### The effects of anticipated emotions on pro-environmental behavior

Past research has highlighted the role anticipated emotions play in predicting pro-environmental behavior, indicating their significance in shaping both intentions and actual behaviors across various contexts. For example, Carrus et al. (2008) explored how anticipated emotions influence individuals’ intentions to engage in ecological activities such as recycling and using public transportation. Their results showed that both anticipated positive emotions (e.g., pride and satisfaction) and negative emotions (e.g., guilt and frustration) positively predicted intentions to engage in these behaviors. Similarly, Kim et al. (2013) found that anticipated regret increased consumers’ likelihood of choosing eco-friendly restaurants. Zhang et al. (2018) reported that anticipated positive emotions enhanced visitors’ intentions to adopt pro-environmental actions within an urban park setting.

Additionally, anticipated emotions have been found influential in organizational contexts. Lu et al. (2020) investigated anticipated pride and guilt among employees, discovering that anticipating pride increased employees’ likelihood of engaging in environmentally friendly behaviors. Conversely, anticipating guilt for failing to adopt sustainable behaviors also motivated employees toward more pro-environmental actions. Further supporting these findings, Mi et al. (2021) demonstrated that anticipated guilt significantly fostered various workplace pro-environmental behaviors, including eco-initiatives, eco-helping, and eco-civic engagement. Shipley and van Riper (2022), in their meta-analysis, further confirmed that anticipated pride and guilt substantially contribute to pro-environmental behavior, with moderate to large effect sizes.

Although existing studies provide compelling evidence for the importance of anticipated emotions for pro-environmental behavior, they typically focus either on a single anticipated emotion or specific behavioral contexts, such as workplaces or consumer decisions. The current study will adopt a valence-based approach by categorizing anticipated emotions into positive and negative types, and investigate their respective influences on individuals’ daily pro-environmental behaviors.

Based on the existing literature, we hypothesize:

*H1:* Anticipated positive emotions positively predict pro-environmental behavior.

*H2:* Anticipated negative emotions positively predict pro-environmental behavior.

### The mediating role of environmental attitudes

Environmental attitudes—reflecting individuals’ values and beliefs about nature and the human–environment relationship—are often regarded as key psychological drivers of ecological behavior (Kaiser et al., 1999). Drawing on AET, which posits that emotional experiences can influence attitudes and subsequently behavior (Weiss and Cropanzano, 1996; Weiss and Beal, 2005), it is plausible that anticipated emotions—defined as affective responses expected in response to potential future events—shape individuals’ environmental attitudes, which in turn influence pro-environmental behavior.

While empirical research on the direct link between anticipated emotions and environmental attitudes remains limited, existing evidence across domains provides compelling support for such a relationship. For instance, anticipated regret has been shown to correlate moderately with attitudes toward precautionary sexual behavior (*r* = 0.33) (Richard et al., 1998). In other research on behaviors such as eating junk food, alcohol consumption, and studying, anticipated positive emotions correlate with attitudes to varying degrees (ranging from 0.23 to 0.75) (Richard et al., 1996). Similarly, anticipated positive emotions, such as excitement or satisfaction, have been found to positively influence attitudes toward fast food brands (Pérez-Villarreal et al., 2019). Recent research has also suggested the impact of anticipated emotions on environmental attitudes. For example, Ibrahim et al. (2021) investigated the role of economically responsible ecotourist attitudes toward staying in local homestays for wildlife conservation, in the relationship between anticipated emotion and the intention to stay in such homestays, revealing that anticipated emotions positively influenced environmental attitudes in this context. Additionally, Mukherjee and Chandra (2022) demonstrated that both anticipated pride and guilt have a positive influence on attitude toward green practice in the workplace. These findings suggest that anticipated emotions can shape environmental attitudes.

The relationship between environmental attitudes and pro-environmental behavior has been extensively documented (Bamberg and Möser, 2007; Kaiser et al., 1999). For example, Kaiser et al. (1999) demonstrated that environmental attitudes are strong predictors of ecological behavior. Liu and Wu (2013) found that environmental attitudes significantly influence individuals’ intentions and subsequent pro-environmental behavior. Research across various domains—such as green purchasing, eco-friendly travel, and sustainable consumer behavior—further underscores the role of environmental attitudes in pro-environmental behaviors. For instance, López-Mosquera et al. (2015) found that environmental attitudes strongly predict behaviors like eco-friendly purchasing and reducing car use. Cheung and To (2019) showed that individuals with stronger environmental consciousness tend to develop more favorable attitudes toward environmental issues and eco-social benefits, which positively influence their green purchasing behavior. Taufique (2020) highlighted that positive attitudes toward the environment promote green consumer behavior. Furthermore, the influence of environmental attitudes on pro-environmental behavior has been confirmed across diverse settings, including both emerging and advanced countries (Adam et al., 2021; Bronfman et al., 2015; Miller and Rice, 2024) and among different population groups, such as workers and students (Cordano and Frieze, 2000; De Leeuw et al., 2015). These studies collectively emphasize the significant role that environmental attitudes play in shaping pro-environmental behavior.

Building on AET and empirical evidence demonstrating the effect of anticipated emotions on environmental attitudes and the influence of environmental attitudes on pro-environmental behavior, we propose that environmental attitudes mediate the relationship between anticipated emotions and pro-environmental behavior.

Specifically, we propose the following hypotheses:

*H3:* Anticipated positive emotions positively predict pro-environmental behavior through the mediating role of environmental attitudes.

*H4:* Anticipated negative emotions positively predict pro-environmental behavior through the mediating role of environmental attitudes.

**Figure 1:**
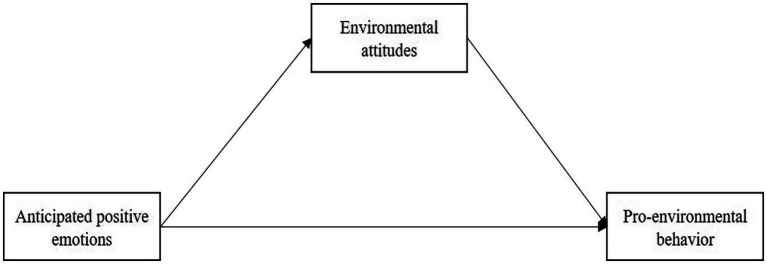
Proposed mediation model for the effects of anticipated positive emotions on pro-environmental behavior.

**Figure 2:**
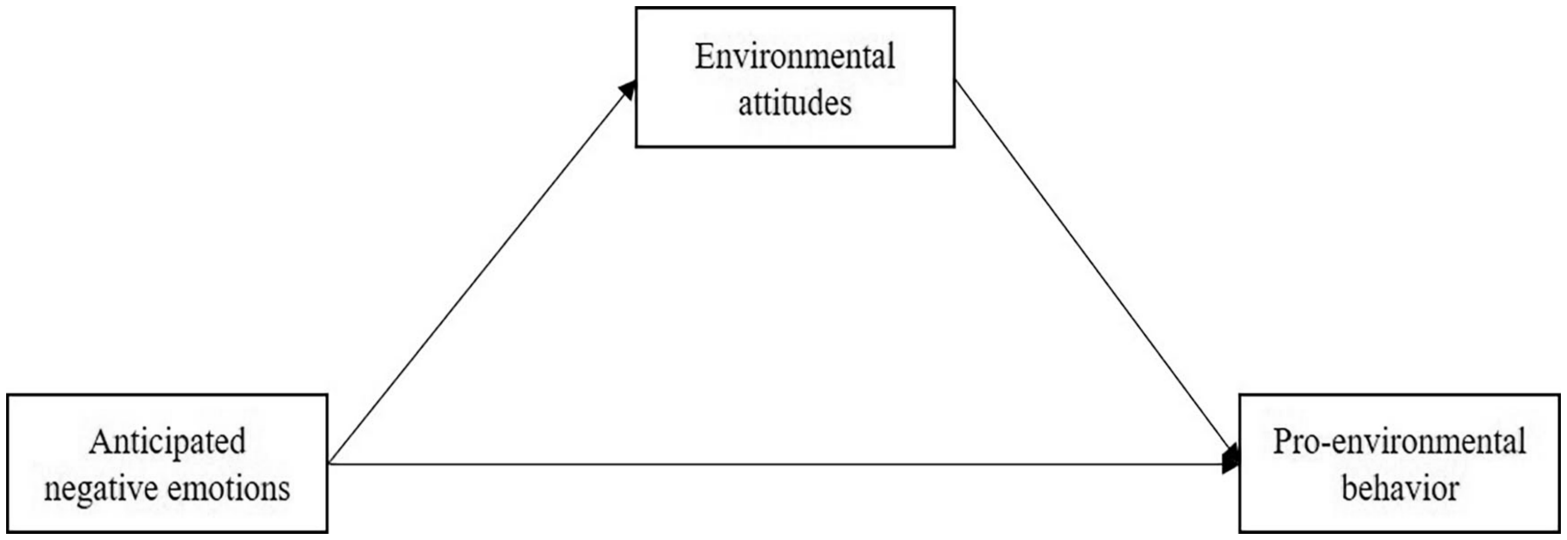
Proposed mediation model for the effects of anticipated negative emotions on pro-environmental behavior.

In sum, this study aims to examine the influence of anticipated positive and negative emotions on pro-environmental behavior and their underlying mechanisms. Based on AET, two separate mediation models were constructed to examine the mechanisms through which anticipated positive and negative emotions influence pro-environmental behavior (Figures 1, 2).

## Materials and methods

### Participants

According to Fritz and MacKinnon (2007), the determination of sample size for mediation analysis is based on the effect size of path coefficients, statistical power, and the testing method. Based on a literature review, the path coefficient from anticipated emotions to environmental attitudes (*α*) is set at 0.14 (small effect), while the path coefficient from environmental attitudes to pro-environmental behavior (*β*) is set at 0.39 (medium effect). Using the bias-corrected nonparametric percentile bootstrap method, with statistical power set at 0.80, the required sample size is 406.

Participants were recruited using the Credamo online platform, a professional data collection service in China comparable to Amazon Mechanical Turk. A total of 500 questionnaires were randomly distributed across China through the Credamo platform, and after excluding responses from participants who did not pass the attention check (e.g., failing to select specific required answers or completing the survey too quickly), 442 valid responses were retained. The number meets the sample size requirement for mediation analysis. The sample was predominantly female (62.22%) and with a wide age range, with the majority aged 26–35 (55.66%). Education levels were relatively high, with 75.34% holding a bachelor’s degree and 17.87% holding a master’s degree. Monthly income varied, with 8.37% earning less than 3,000 CNY, 22.4% between 5,001 and 8,000 CNY, 21.27% between 8,001 and 10,000 CNY, and 33.94% exceeding 10,000 CNY. The majority resided in urban areas (95.48%). Detailed demographic information is presented in Table 1.

**Table 1:** Demographics profiles of participants.

Demographics		Frequencies	Percentages (%)
Gender	Female	275	62.22
	Male	167	37.78
Age	25 and under	97	21.95
	26–35	246	55.66
	36–45	75	16.97
	46 and over	24	5.43
Education level	High school or below	7	1.58
	Associate degree	23	5.20
	Bachelor’s degree	333	75.34
	Master’s degree	79	17.87
Monthly income	Lower than 3,000 CNY	62	14.03
	3,000–5,000 CNY	37	8.37
	5,001–8,000 CNY	99	22.40
	8,001–10,000 CNY	94	21.27
	More than 10,000 CNY	150	33.94
Residence type	Urban	422	95.48
	Rural	20	4.52

CNY, Chinese Yuan.

This study was approved by the Medical Ethics Committee of the Northwest University, and written informed consent was obtained from the participants.

### Measures

This study specifically measured the following variables: (1) anticipated positive and negative emotions, (2) environmental attitudes, (3) pro-environmental behavior, and (4) demographic variables.

### Anticipated positive and negative emotions

Baumgartner et al. (2008) developed an 8-item scale measuring anticipated positive and negative emotions. Feng et al. (2015) revised the Chinese version of the anticipated emotions scale. This scale requires participants to imagine themselves in future positive or negative situations and evaluate their potential positive or negative emotional responses.

In the present study, we further adapted this measure to assess anticipated emotions in the context of pro-environmental behavior, employing a valence-based approach using two imagined environmental scenarios. Participants were presented with two imagined environmental scenarios in a fixed order. In the first scenario, participants were instructed to imagine a future where environmental conditions had significantly improved due to effective environmental protection and planning. They then rated their anticipated emotional responses across four positive dimensions: relaxation, satisfaction, happiness, and pride. In the second scenario, participants imagined a future in which environmental conditions had severely deteriorated as a result of inadequate environmental protection. They then evaluated four negative emotions: disappointment, annoyance, regret, and anger. Each item was rated on a 7-point Likert scale ranging from 1 (strongly disagree) to 7 (strongly agree), reflecting the extent to which each emotion matched their expected feelings in the described scenarios. The scale demonstrated acceptable internal consistency, with Cronbach’s alpha coefficients of 0.72 for positive emotions and 0.70 for negative emotions.

### Environmental attitudes

The New Ecological Paradigm (NEP) scale is extensively employed for environmental attitudes due to its capture of cognitive beliefs about the human–nature relationship (Dunlap et al., 2000; Hawcroft and Milfont, 2010; Kaltenborn et al., 2009). In this study, we adopt a cognitive perspective on environmental attitudes, aligning with AET, which views attitudes—such as job satisfaction—as cognitively dominant evaluative judgments that correspond to beliefs and cognitive evaluations (Weiss and Cropanzano, 1996). This cognitive focus is appropriate for examining how anticipated emotions influence belief-based evaluations of the human–environment relationship. The Chinese version of the NEP scale, revised by Wu et al. (2012), was used to assess environmental attitudes. The scale includes 15 items, such as “Humans have the right to modify the natural environment to satisfy their desires” and “Human interference with nature often produces disastrous consequences.” A 5-point Likert scale was used, with responses ranging from 1 (strongly disagree) to 5 (strongly agree). In this study, the scale demonstrated acceptable reliability with a Cronbach’s alpha coefficient of 0.76.

### Pro-environmental behavior

The pro-environmental behavior scale was adopted from Liu and Wu (2013) who developed it to measure both public and private domain pro-environmental behaviors. This scale has demonstrated good reliability and validity. It consists of 11 items, including examples such as: “Advising others to stop behaviors that harm the environment” “Reusing plastic bags” and “Turning off lights or fans when leaving the room if no one is there.” Participants were asked to rate whether they engaged in these behaviors over the past year, using a 5-point Likert scale ranging from 1 (Never) to 5 (Often). In this study, the scale showed acceptable reliability, with a Cronbach’s alpha coefficient of 0.77.

### Demographic variables

The study also collected participants’ demographic variables, including gender, age, type of residential area, education level, and income level. These demographic variables were included as control variables in the statistical analysis.

### Statistical analysis

The statistical analysis was conducted using SPSS 26. First, common method bias was assessed. Next, descriptive statistics and correlation analyses were performed. Subsequently, the research hypotheses were tested using the PROCESS macro 4.2 for SPSS (Hayes, 2017).

## Results

### Common method bias analysis

To assess the potential common method bias resulting from self-reported data, this study conducted a Harman’s single-factor analysis (Podsakoff et al., 2003). The results indicated that there were nine factors with eigenvalues greater than 1. The first factor explained 15.05% of the total variance, which is below the 40% threshold, suggesting that common method bias is not a concern in this study.

### Descriptive statistics and correlation analysis

The results of the descriptive statistics and partial correlation analysis are presented in Table 2. The findings indicate that pro-environmental behavior is significantly positively correlated with anticipated positive emotions, anticipated negative emotions, and environmental attitudes. Additionally, environmental attitudes show a significant positive correlation with both anticipated positive emotions and anticipated negative emotions. Furthermore, anticipated positive emotions are significantly positively correlated with anticipated negative emotions.

**Table 2:** Descriptive statistics and correlations among study variables after controlling for demographic factors.

Covariates	Variables	*M*	SD	PEB	APE	ANE	EA
Age, sex, education, income, and residence	PEB	4.14	0.48	1			
	APE	6.25	0.64	0.18^***^	1		
	ANE	5.79	0.73	0.14^**^	0.30^***^	1	
	EA	4.37	0.37	0.26^***^	0.18^***^	0.26^***^	1

APE, anticipated positive emotions; ANE, anticipated negative emotions; EA, environmental attitudes; PEB, pro-environmental behavior. ^**^p < 0.01, ^***^p < 0.001.

### The effect of anticipated positive emotions on pro-environmental behavior and the mediating role of environmental attitudes

Mediation analysis was conducted using PROCESS Model 4 to examine the relationship between anticipated positive emotions, environmental attitudes, and pro-environmental behavior, controlling for demographic variables such as age, gender, education level, income, and type of residence. First, the total effects of anticipated positive emotions on pro-environmental behavior was tested. The regression model was significant, *F*(6, 435) = 8.05, *p* < 0.001, adjusted *R*^2^ = 0.09. Anticipated positive emotions positively predicted pro-environmental behavior, *b* = 0.13, *p* < 0.001, 95% CI [0.06, 0.20]. This finding confirmed Hypothesis 1, indicating that more intense anticipated positive emotions were associated with increased engagement in pro-environmental actions.

**Figure 3:**
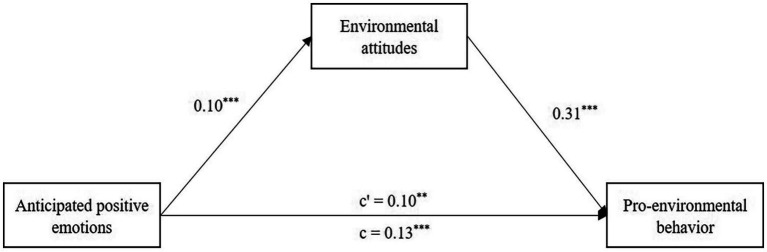
Mediation model of the relationship between anticipated positive emotions and pro-environmental behavior through environmental attitudes. ^**^p < 0.01, ^***^p < 0.001.

To test Hypothesis 3, we examined the mediation effect of environmental attitudes in the relationship between anticipated positive emotions and pro-environmental behavior. Anticipated positive emotions positively predicted environmental attitudes, *b* = 0.10, *p* < 0.001, 95% CI [0.05, 0.15], and environmental attitudes positively predicted pro-environmental behavior, *b* = 0.31, *p* < 0.001, 95% CI [0.19, 0.43]. These results are detailed in Table 3. Using a bias-corrected nonparametric percentile bootstrap method (with 5,000 times of sampling), the direct effect of anticipated positive emotions on pro-environmental behavior was significant, effect = 0.10, *t* = 2.90, *p* < 0.01, 95% CI [0.03, 0.16], and the indirect effect of anticipated positive emotions on pro-environmental behavior through environmental attitudes was also significant, effect = 0.03, 95% CI [0.01, 0.06]. These findings confirm Hypothesis 3, suggesting partial mediation: anticipated positive emotions influence pro-environmental behavior both directly and indirectly through environmental attitudes (Figure 3).

**Table 3:** Model coefficients for the anticipated positive emotion mediation analysis.

Variables	EA				PEB			
	*b*	SE	*p*	95% CI	*b*	SE	*p*	95% CI
APE	0.10	0.03	< 0.001	[0.05, 0.15]	0.10	0.03	<0.01	[0.03, 0.16]
EA	–	–	–	–	0.31	0.06	<0.001	[0.19, 0.43]
Age	−0.00	0.00	0.189	[−0.01, 0.00]	−0.00	0.00	0.208	[−0.01, 0.00]
Sex	−0.04	0.04	0.287	[−0.11, 0.03]	0.09	0.04	<0.05	[0.00, 0.17]
Education	−0.06	0.03	0.075	[−0.13, 0.01]	−0.02	0.04	0.699	[−0.10, 0.07]
Income	0.06	0.01	< 0.001	[0.03, 0.09]	0.08	0.02	<0.001	[0.04, 0.11]
Residence	−0.14	0.08	0.102	[−0.30, 0.03]	−0.03	0.11	0.798	[−0.24, 0.18]
	Adjusted *R*^2^ = 0.08				Adjusted *R*^2^ = 0.14			
	*F*(6, 435) = 7.21, *p* < 0.001				*F*(7, 434) = 11.07, *p* < 0.001			

APE, anticipated positive emotions; EA, environmental attitudes; PEB, pro-environmental behavior.

### The effect of anticipated negative emotions on pro-environmental behavior and the mediating role of environmental attitudes

We performed mediation analysis using PROCESS Model 4 to examine the relationship between anticipated negative emotions, environmental attitudes, and pro-environmental behavior, controlling for age, gender, education level, income, and type of residence. Initially, we assessed the total effect of anticipated negative emotions on pro-environmental behavior. The regression model was significant, *F*(6, 435) = 7.15, *p* < 0.001, adjusted *R*^2^ = 0.08. Anticipated negative emotions significantly predicted pro-environmental behavior, *b* = 0.09, *p* < 0.01, 95% CI [0.03, 0.15], confirming Hypothesis 2 and indicating that stronger anticipated negative emotions were associated with greater engagement in environmentally responsible behavior.

**Figure 4:**
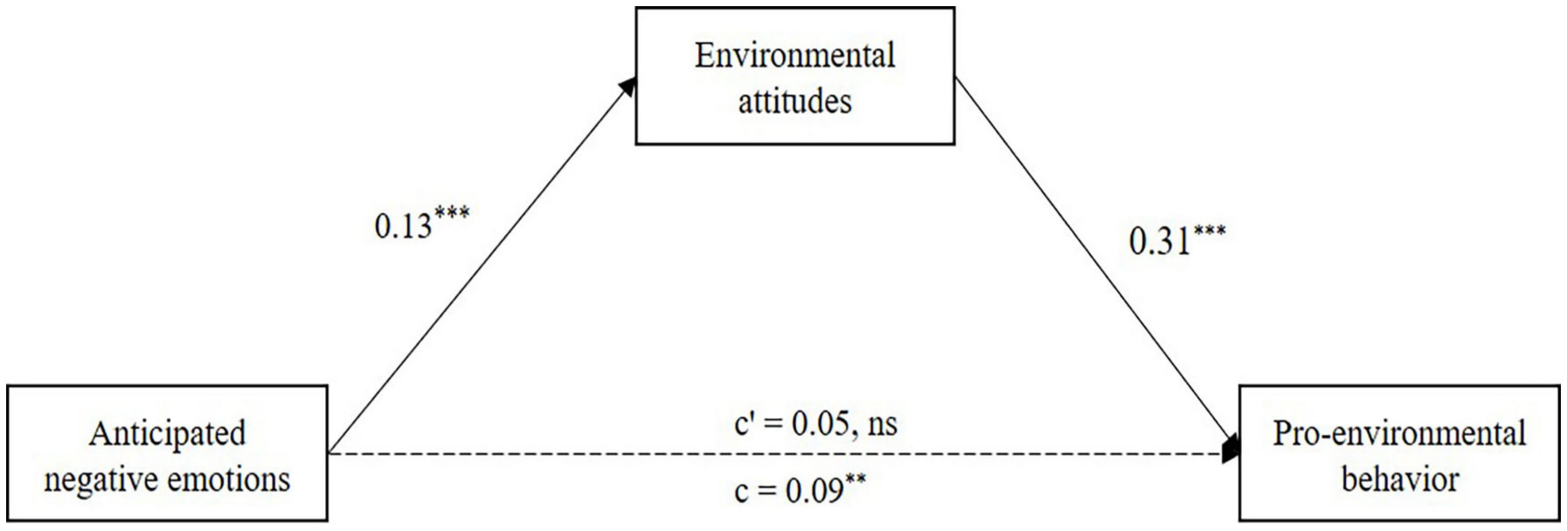
Mediation model of the relationship between anticipated negative emotions and pro-environmental behavior through environmental attitudes. ^**^p < 0.01, ^***^p < 0.001, ns, not significant.

To evaluate Hypothesis 4, we assessed the mediating role of environmental attitudes in the relationship between anticipated negative emotions and pro-environmental behavior. The results showed that anticipated negative emotions positively predicted environmental attitudes, *b* = 0.13, *p* < 0.001, 95% CI [0.08, 0.17], and environmental attitudes positively predicted pro-environmental behavior, *b* = 0.31, *p* < 0.001, 95% CI [0.19, 0.43]. These results are detailed in Table 4. Using a bias-corrected nonparametric percentile bootstrap method (with 5,000 resamples), the direct effect of anticipated negative emotions on pro-environmental behavior was non-significant, effect = 0.05, *t* = 1.71, *p* = 0.08, 95% CI [−0.01, 0.11], but the indirect effect through environmental attitudes was significant, effect = 0.04, 95% CI [0.02, 0.07]. These findings confirm Hypothesis 4, suggesting full mediation: anticipated negative emotions influence pro-environmental behavior indirectly through environmental attitudes (Figure 4).

**Table 4:** Model coefficients for the anticipated negative emotion mediation analysis.

Variables	EA				PEB			
	*b*	SE	*p*	95% CI	*b*	SE	*p*	95% CI
ANE	0.13	0.22	<0.001	[0.08, 0.17]	0.05	0.03	0.088	[−0.01, 0.11]
EA	–	–	–	–	0.31	0.06	<0.001	[0.19, 0.43]
Age	−0.00	0.00	0.153	[−0.01, 0.00]	−0.00	0.00	0.181	[−0.01, 0.00]
Sex	−0.03	0.04	0.335	[−0.10, 0.03]	0.09	0.04	<0.05	[0.01, 0.18]
Education	−0.07	0.03	0.054	[−0.13, 0.00]	−0.02	0.04	0.626	[−0.11, 0.06]
Income	0.06	0.01	<0.001	[0.04, 0.09]	0.07	0.02	<0.001	[0.04, 0.11]
Residence	−0.12	0.08	0.147	[−0.28, 0.04]	−0.03	0.11	0.763	[−0.24, 0.18]
	Adjusted *R*^2^ = 0.11				Adjusted *R*^2^ = 0.13			
	*F*(6, 435) = 10.09, *p* < 0.001				*F*(7, 434) = 10.16, *p* < 0.001			

ANE, anticipated negative emotions; EA, environmental attitudes; PEB, pro-environmental behavior.

## Discussion

This study examined the effects of anticipated positive and negative emotions on pro-environmental behavior and the underlying mechanisms driving these effects. The findings show that both anticipated positive and negative emotions have a positive impact on pro-environmental behavior. Anticipated positive emotions not only directly affect pro-environmental behavior but also exert an indirect influence through environmental attitudes. In contrast, anticipated negative emotions influence pro-environmental behavior fully through environmental attitudes.

Both anticipated positive and negative emotions were found to positively predict pro-environmental behavior, thus validating Hypotheses 1 and 2. These results are consistent with prior research showing the positive impact of anticipated emotions on pro-environmental behavior (Kim et al., 2013; Lu et al., 2020; Zhang et al., 2018). For example, anticipated positive emotions have been shown to motivate urban park visitors to engage in pro-environmental behavior (Zhang et al., 2018); both anticipated positive and negative emotions (e.g., pride and guilt) positively affected employees’ pro-environmental behavior (Lu et al., 2020). Our findings also align with meta-analysis research demonstrating the positive effects of anticipated emotions on pro-environmental behavior (Rivis et al., 2009; Sandberg and Conner, 2008). These findings support the argument that anticipated emotions can guide behavior by eliciting positive or negative feelings associated with their behavioral choices (Richard et al., 1996).

The Emotion-as-Feedback System Theory offers further insight, suggesting that anticipated emotional outcomes act as feedback signals that motivate individuals to engage in behaviors leading to desired emotions and avoid those that result in undesirable emotions (DeWall et al., 2016; Richard et al., 1996). In the context of pro-environmental behavior, the anticipation of positive emotions from engaging in pro-environmental behavior encourages individuals to act in alignment with ecological goals. On the other hand, anticipated negative emotions arising from inaction in pro-environmental behavior may drive individuals to adopt more eco-friendly behaviors to avoid these negative emotional consequences.

Our study found that both anticipated positive and negative emotions influence pro-environmental behavior through environmental attitudes. These results support Hypotheses 3 and 4 and are consistent with AET, highlighting that anticipated emotions play a crucial role in shaping environmental attitudes, which in turn influence pro-environmental behavior. While prior research has demonstrated the role of anticipated emotions in predicting pro-environmental behavior, most studies have examined these effects within the TPB framework without fully explaining the underlying mechanisms of anticipated emotions (Lu et al., 2020; Zhang et al., 2018). Our study advances this work by integrating AET, demonstrating the mediating role of environmental attitudes in the relationship between anticipated emotions and pro-environmental behavior. In this study, environmental attitudes were assessed using the New Ecological Paradigm scale, which measures individuals’ perceptions of the relationship between humans and the environment. These findings suggest that anticipated emotions may affect individuals’ views on the human–nature relationship, thereby influencing their pro-environmental behavior. This finding provides deeper insights into the psychological mechanisms through which emotional factors operate within the framework of the TPB.

Notably, the present study identified distinct mediating mechanisms underlying the effects of anticipated positive and negative emotions on pro-environmental behavior. Anticipated positive emotions influenced pro-environmental behavior through both direct and indirect pathways. The indirect pathway indicates that individuals first appraise their relationship with the environment, as reflected in their environmental attitudes, before engaging in pro-environmental behavior. The direct pathway underscores the motivational role of positive emotions. According to the motivational theory of emotions (Frijda, 1986), positive emotions activate approach motivation, prompting individuals to pursue behaviors associated with desirable outcomes. Accordingly, the anticipation of positive emotions resulting from pro-environmental behavior may serve as an intrinsic motivator, directly enhancing engagement in such behaviors. In contrast, anticipated negative emotions were found to affect pro-environmental behavior solely through an indirect pathway mediated by environmental attitudes. The absence of a direct effect may be attributable to the avoidance-oriented motivational function of negative emotions. Anticipated negative emotions represent the expected aversive affective states resulting from the failure to engage in pro-environmental behavior, thereby motivating individuals to adopt behaviors that align with pro-environmental goals in order to mitigate these anticipated feelings. Furthermore, the lack of a direct association may also be explained by coping strategies for negative emotions, such as attentional redirection or psychological distancing from adverse environmental outcomes (Lazarus, 1991). These coping mechanisms may reinforce the evaluative process of environmental attitudes, ultimately promoting pro-environmental behavioral engagement.

These findings implicate that pro-environmental behavior can be enhanced by inducing anticipated emotions. Experimental methods for inducing such emotions include affective forecasting, in which individuals imagine future emotional responses such as pride from engaging in eco-friendly actions, and reflective writing, in which people write about upcoming decisions that evoke positive or negative feelings. Both methods have been demonstrated to elicit anticipated emotions and promote pro-environmental intentions and actions (Bergquist et al., 2022; Schneider et al., 2017). Recent meta-analytic evidence indicates that pride exerts a stronger and more sustained effect than guilt (Shipley and van Riper, 2022), thereby supporting interventions focused on positive emotions. Furthermore, it is feasible to train individuals to anticipate positive emotions. For instance, guided episodic thinking about positive future events has been shown to elevate anticipated pleasure, which can motivate pro-environmental behaviors through enhanced emotional forecasting (Hallford et al., 2022).

Although this study makes an important contribution, it also has several limitations. First, the cross-sectional design of this study precludes causal inferences and limits our understanding of whether anticipated positive and negative emotions remain stable over time or diminish. Longitudinal studies are needed to examine the persistence of these emotional influences on pro-environmental behavior and to track changes in environmental attitudes over extended periods. Second, the fixed order of the emotional scenarios (positive followed by negative) may have influenced participants’ responses due to potential order effects. This may have influenced participants’ ratings of anticipated emotions, thereby posing a potential threat to the internal validity of the findings. Therefore, future research should address this issue by randomizing the order in which the scenarios are presented. Third, while the sample was representative of multiple provinces in China, the majority of participants were urban residents, with fewer rural residents. Therefore, future studies should collect more data from rural populations to further validate the findings and explore whether there are differences between urban and rural groups. Fourth, this study demonstrated that anticipated emotions influence pro-environmental behavior through the mediating role of environmental attitudes. However, previous research has indicated that the effect of environmental attitudes on pro-environmental behavior may be moderated by various factors, such as demographic variables (e.g., educational attainment; Bamberg and Möser, 2007) and personal costs (Wyss et al., 2022). Therefore, future studies should investigate the moderating roles of these factors to further elucidate the boundary conditions under which anticipated emotions affect pro-environmental behavior.

## Conclusion

This study investigated how anticipated positive and negative emotions affect pro-environmental behavior through the theoretical lens of affective events theory. The results demonstrated that both types of emotions influence pro-environmental behavior, with environmental attitudes partially mediating the effects of anticipated positive emotions and fully mediating the effects of anticipated negative emotions. These findings not only confirm the impact of both anticipated positive and negative emotions on pro-environmental behavior but also reveal important similarities and differences in the underlying mechanisms. Together, they provide both theoretical insights into the emotional pathways influencing pro-environmental behavior and practical implications such as emotional interventions for promoting such behaviors.

## Data Availability

The raw data supporting the conclusions of this article will be made available by the authors, without undue reservation.
